# Biodiversity science in China

**DOI:** 10.1093/nsr/nwab097

**Published:** 2021-07-26

**Authors:** Peter H Raven

**Affiliations:** Missouri Botanical Garden, USA

Progress in the systematic investigation of the biota of China, particularly over the past 50 years, has been most impressive. A century ago, foreign investigators were conducting most of these studies, but starting in the 1920s, the effort moved gradually to become an international one. Previously slowed by war and the ‘Cultural Revolution’, activities in this field began to grow in the 1970s, and have continued to expand subsequently. The results from hundreds of investigators in dozens of institutions, often with many foreign collaborators, are shown clearly in the detailed review published here [1]. These include not only inventories for China as a whole and for many of its regions, but also analyses of the evolutionary and ecological processes responsible for the existing patterns.

Judged by the proportion of species in groups that we know well enough to make comparisons meaningful, China is second only to Brazil in national species richness. Approximately 9% of the world's species probably occur in China and perhaps slightly more than 10% in Brazil. This would indicate that ∼180 000 of the species known globally, and conservatively perhaps 2 million species of eukaryotic organisms known and unknown, actually occur in China, depending on what the global total actually may turn out to be.

As we reviewed in two recent articles [2,3], the only substantial groups of organisms that are well enough known to use in geographical comparisons are terrestrial vertebrates, vascular plants and a few others such as butterflies and mosquitoes. As the accompanying review [1] shows, there are a few other groups in China (e.g. bryophytes, lichens) currently well enough known to warrant trying hard to obtain a more-or-less complete picture for the country. When it comes to those groups that we know very little about globally, such as nematodes (for which we have named 25 000 species globally), mites (for which we have named 64 000 globally) and fungi (100 000 named globally), extinction is proceeding so rapidly that we must adopt different strategies. For the first two of these, experts estimate that more than 1 000 000 living species exist, and for the third, 2.2–2.7 million species. Relationships with well-known groups suggest that China might have something approaching one-tenth of these latter totals of species.

In order to be able, efficiently, to delineate patterns of distribution in groups such as the three just mentioned, it would be ideal to agree globally on appropriate methods of sampling for China and elsewhere. In a world where habitat destruction and climate change have launched what most scientists consider the sixth major extinction event, we will have to move rapidly or lose the opportunity forever. One hopes that China, with the kind of vital energy shown in the results presented in the review in this volume, will become a leader in working out what to do to break away from Linnaean assumptions most usefully. On top of these critical factors, members of all three groups just mentioned are both environmentally and economically highly important both in China and throughout the world, and focused study is badly needed on those accounts also.

In a global context, China is of vital importance both because of its biological richness and because it is such a significant refuge for plants and animals, many of which occurred throughout the northern hemisphere in Paleogene time. The best known of these is *Ginkgo biloba*, which occurred through much of Eurasia and North America at one time, but there are many more examples of such survivors at all taxonomic levels. In addition, parts of China are ‘global hotspots’, areas of rapid speciation and/or unusually high levels of survival.

On a side note, one hopes that China will examine the evidence for possible harmful environmental or other effects of crops altered by modern methods. With hundreds of millions of animals and people using these methods in the United States and not a single harmful impact demonstrated, the evidence for harm seems to be absent. Consequently, it does seem time to put aside the political and other considerations that are depriving the Chinese people of crops as productive as they could be, and move on.

Summarizing, then, China has clearly grown to become a world leader in the study of biodiversity, with hundreds of scientists and dozens of first-rate research institutions contributing substantially to our overall effort. With strong international cooperation, we can decide best what will be most important to do in this age of rapid, mass extinction. The results we do achieve will in many cases, unfortunately, be the only traces of the abundant life that exists today.

***Conflict of interest statement.*** None declared.
